# Genetics Insight for COVID-19 Susceptibility and Severity: A Review

**DOI:** 10.3389/fimmu.2021.622176

**Published:** 2021-04-01

**Authors:** Ingrid Fricke-Galindo, Ramcés Falfán-Valencia

**Affiliations:** HLA Laboratory, Instituto Nacional de Enfermedades Respiratorias Ismael Cosío Villegas, Mexico City, Mexico

**Keywords:** COVID-19, genetics, ACE2, HLA, SNV, SARS-CoV-2

## Abstract

Coronavirus disease (COVID-19) presents a broad spectrum of clinical manifestations ranging from an asymptomatic to a severe clinical course. The host genetic background influence on the susceptibility and outcome of multiples infectious diseases has been previously reported. Herein, we aimed to describe relevant identified genetic variants and those potentially related to the inter-individual variability of COVID-19 susceptibility and/or severity considering the physiopathological pathway of the disease The HLA-A*25:01, -B*15:27, -B*46:01, -C*01:02, and -C*07:29 alleles have been associated with COVID-19 susceptibility; while HLA-A*02:02, -B*15:03, and -C*12:03 have been identified as low-risk alleles. Variants in cytokine genes such as *IL1B*, *IL1R1*, *IL1RN*, *IL6*, *IL17A*, *FCGR2A*, and *TNF* could be related to disease susceptibility and cytokine storm, and/or COVID-19 complications (e.g., venous thrombosis). Several variants in *ACE2* and *TMPRSS2* affecting the expression of the receptors related to COVID-19 have been associated with the disease susceptibility and risk factors. Finally, two GWAS have identified the *loci* 3p21.31 (*LZTFL1*, *SLC6A20*, *CCR9*, *FYCO1*, *CXCR6*, and *XCR1*) and 9q34.2 (*ABO*) with COVID-19 severity. Heterogeneous results in the association of genetic variants with COVID-19 susceptibility and severity were observed. The mechanism of identified risk-genes and studies in different populations are still warranted.

## Introduction

The Coronavirus Disease 2019 (COVID-19) is a severe respiratory and systemic disease caused by the novel Severe Acute Respiratory Syndrome Coronavirus 2 (SARS-CoV-2). The first cases of the new disease were reported in Wuhan, Hubei Province of China, and it has spread quickly to the rest of the worldwide population. Until March 3, 2021, countries have reported to the World Health Organization (WHO) a total of 114,428,211 confirmed cases of COVID-19, a cipher that unfortunately includes 2,543,755 deaths (1).

**Figure 1:**
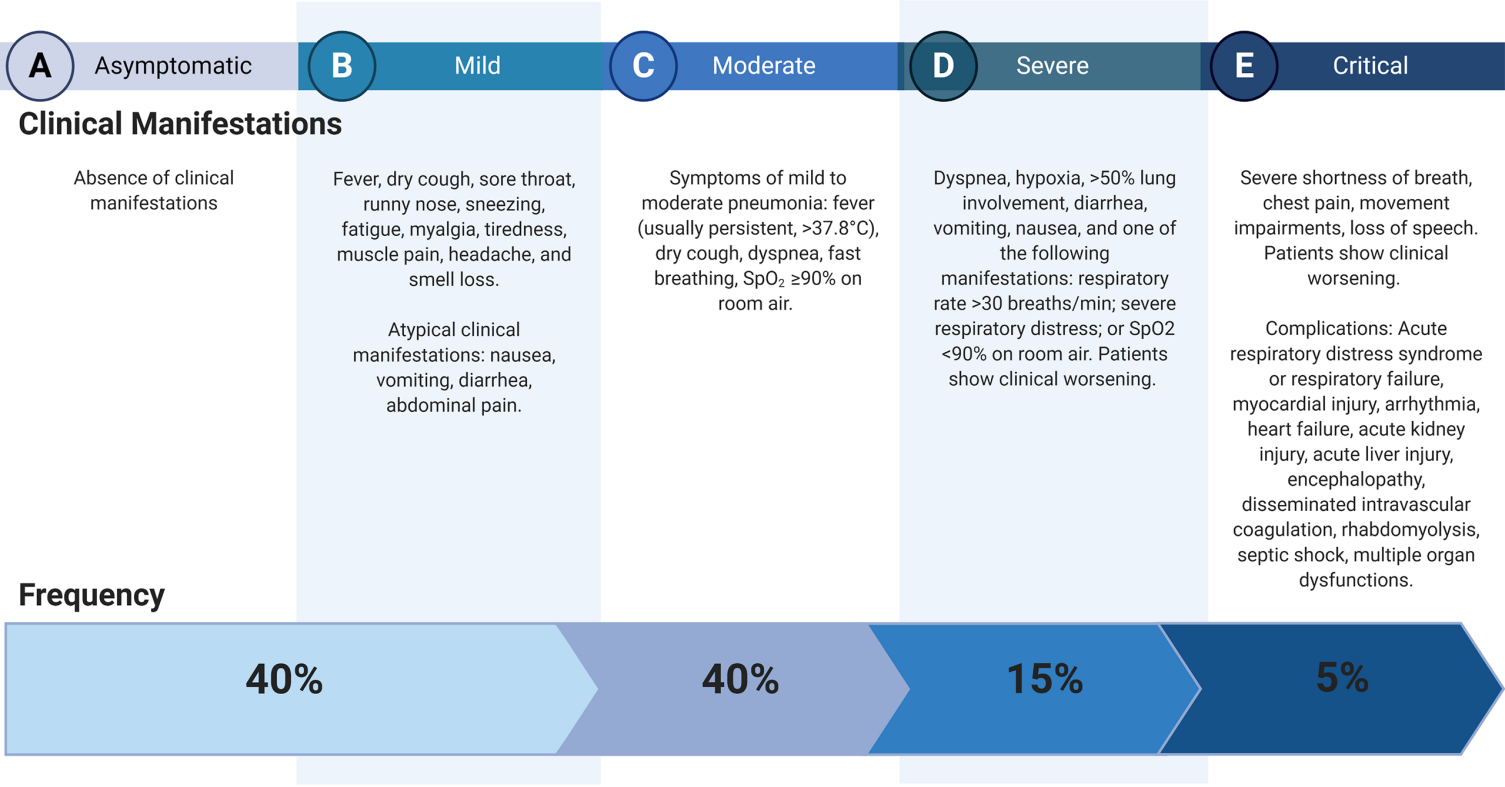
Clinical courses of COVID-19. Data (2) and Clinical management of COVID-19, interim guidance (3). Created with BioRender.com.

COVID-19 is a complex disease that presents a broad spectrum of clinical manifestations ranging from an asymptomatic to a severe clinical course (**Figure 1**). This infection is considered a systemic disease involving the cardiovascular, respiratory, gastrointestinal, neurological, hematopoietic, and immune systems (4–6). The mortality rate reported ranges between 1-7% (7); respiratory failure, septic shock, multiorgan failure, and cardiac arrest are considered the leading causes of death (8, 9).

Acute Respiratory Distress Syndrome (ARDS) is developed by 41.8% of patients with COVID-19, mainly in those with comorbidities such as diabetes mellitus, hypertension, cardiovascular disease, and chronic kidney disease (10). Myocardial injury (including acute coronary syndrome, myocarditis, heart failure, hypotension, shock, and sepsis) is strongly associated with death and severe cases of the COVID-19 (9, 11), and it has been explained by the presence of Angiotensin-Converting Enzyme 2 (ACE2) in myocardial cells and the cytokine storm produced after the SARS-CoV-2 infection (12). COVID-19 has also been associated with coagulation abnormalities (e.g., disseminated intravascular coagulation or thrombotic microangiopathy) related to a massive release of plasminogen activators as a product of inflammation-induced endothelial cell injury (13). Other complications of COVID-19, such as acute kidney injury, co-infection with another pathogen, thromboembolism, and/or multiorgan failure, have been reported (3, 9).

Given the broad spectrum of COVID-19 clinical course and complications, identifying risk factors that could predict the disease’s severity would improve the infected patients’ outcome. In this sense, older age, smoking, hypertension, diabetes mellitus, cardiac disease, chronic lung disease, and cancer have been associated with COVID-19 severity and death (3, 14). Nevertheless, these conditions do not explain the total cases of the severity and mortality of COVID-19; therefore, genetic variations influencing the clinical outcome could be considered. Also, regional differences in the frequencies of some COVID-19 clinical manifestations have been observed. For instance, fever and dyspnea were more frequent in patients from Wuhan (91.7% and 21.1%, respectively) than in patients from other regions of China (78.1% and 3.80%) (15).

Moreover, olfactory disturbance or loss of smell seems to be a common symptom among Europeans (16) and Americans from the United States (17), but not for Asians (18). Such variations could be due to demographic, cultural, and dietary habit differences, but genetic variations exist worldwide.

### SARS-CoV-2 Protein Interaction and Immune Response

The knowledge of virus interaction with human proteins and the immune mechanism against the infection is crucial to identifying target genes to study the susceptibility and severity of COVID-19. The SARS-CoV-2 infects alveolar epithelial cells through receptor-mediated endocytosis. The SARS-CoV-2 spike protein (S) binds to the ACE2 receptor, which is expressed in several organs, including the lung, heart, kidney, and intestine (19). Following the receptor binding, the virus enters the host cell cytosol through acid-dependent proteolytic cleavage of the S protein, in which some proteases, including Transmembrane Serine Protease 2 (TMPRSS2) and Cathepsin L (CTSL), cleave to S domains to mediate membrane fusion and virus infectivity (20, 21).

The innate immune response to SARS-CoV-2 infection comprises a mechanical barrier including cells of the pulmonary epithelium and tissue-resident macrophages and dendritic cells. Both immune cells express pattern recognition receptors which can detect Pathogen-Associated Molecular Patterns (PAMPs) and Damage-Associated Molecular Patterns (DAMPs) (22), which triggers the activation of cytoplasmic NOD‐Like Receptor family and Pyrin domain-containing 3 protein (NLRP3) inflammasome pathway (23). The inflammasome activation in macrophages, epithelial cells, and endothelial cells releases pro‐inflammatory cytokines, Interleukin (IL)‐1β and IL‐18, which produce neutrophilia and leukopenia, contributing to the pathogenic inflammation responsible for the severity of symptoms of COVID‐19 (24, 25). Besides, Toll-Like Receptor (TLR)3, TLR7, TLR8, and TLR9, sensing viral RNA, activate the Nuclear Factor kappa B (NF-κB) pathway and a high number of pro-inflammatory cytokines with a significant role in initiating virus-induced inflammation (26). The increased secretion of the pro-inflammatory cytokines and chemokines IL-6, Interferon-gamma (IFN-γ), Monocyte Chemoattractant Protein-1 (MCP-1), and IFN-γ-induced Protein 10 (IP-10) attracts immune cells, notably monocytes and T lymphocytes, but not neutrophils, from the blood into the infected site, explaining the lymphopenia and the increased neutrophil-lymphocyte ratio seen in around 80% of patients with SARS-CoV-2 infection (27).

Commonly, recruited cells scrub the infection in the lung, the immune response subsidies, and the patient recovers. Nevertheless, in patients with severe COVID-19, a dysfunctional immune response occurs, triggering a cytokine storm, in which an increase of IL-2, IL-6, IL-7, IL-10, Granulocyte Colony-Stimulating Factor (G-CSF), IP-10, MCP-1, Macrophage Inflammatory Protein 1α (MIP-1α) and Tumor Necrosis Factor-alpha (TNF-α) in plasma blood levels are observed (27, 28). Although the mechanism leading to the cytokine storm remains unknown, the interferon signaling pathway’s antagonists have been considered (27). Levels of cytokines can explain some of the COVID-19 complications, such as septic shock and multiorgan failure due to TNF-α increase; moreover, cytokine storm is also found in older patients and those with comorbidities, which are considered risk factors for the disease complication (11, 27).

As an antiviral mechanism, antigen-presenting cells are involved in presenting antigenic peptides through the Major Histocompatibility Complex (MHC) class I and class II molecules to CD8+ and CD4+ T cells (29). Both T and B cell responses against SARS-CoV-2 can be detected in the blood around 1 week after the onset of COVID-19 symptoms (27). CD8+ T cells are activated, start cell division and clonal expansion, and develop virus-specific effectors and memory T cells to cause lysis to the infected cells. B cells can be activated directly by the virus recognition and by the interaction with CD4+ T cells. Immunoglobulin (Ig) M antibody can be detected at the early stages of infection, while IgG antibodies are then produced for lifelong immunity (25).

Given the well-known influence of the host genetic background in the susceptibility and outcome of multiples infectious diseases, including coronavirus infections (30), we aimed to describe relevant identified genetic variants and those potentially related to the inter-individual variability of COVID-19 susceptibility and/or severity considering the physiopathological pathway of the disease.

**Figure 2:**
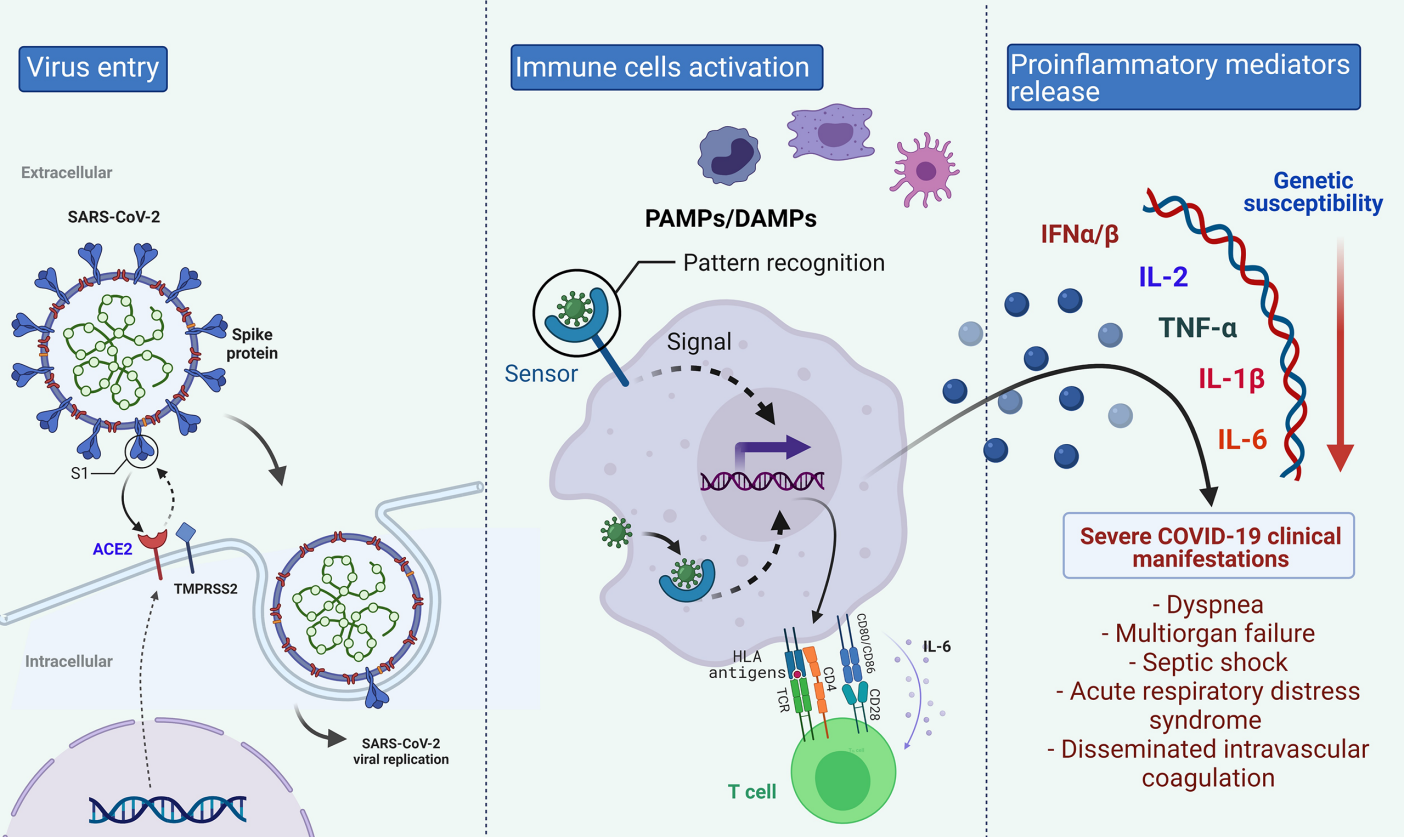
Biological Pathway of COVID-19 in which different genes could be implicated in the disease’s differential clinical outcome. Created with BioRender.com.

The clinical outcome variation to life-threatening pathogens shows the functional genetic diversity of the immune response, differences in the pathogen’s interaction with host proteins, and/complex gene-gene and gene-environment interactions (28,29). Therefore, the genes described for COVID-19 susceptibility and severity were classified if they were related to the immune system, to the SARS-CoV-2 receptor, or other genes reported to be associated with the disease susceptibility or its complications (**Figure 2**).

We performed a literature search of relevant articles in scientific databases (i.e., PubMed, WHO, GenBank, dbSNP, HUGO Gene Nomenclature Committee) from July 2020 to February 2021. The quest includes the following search terms: ‘COVID-19’, ‘genetics’, ‘genomics’, ‘HLA’, ‘disease susceptibility’, ‘ACE2’, ‘disseminated intravascular coagulation’, ‘venous thrombosis’. According to the data relevance, articles were selected, and those studying the SARS-CoV-2 genome and preprints were excluded. Information was analyzed and summarized; thus, the analysis and conclusions of those results are reported in the present review.

## Genetic Variants in the Study of COVID-19 Susceptibility and Severity

### Variants in Genes Related to the Immune System

#### Human Leukocyte Antigens Gene Complex

The immune system is a complex and effective defense mechanism against pathogens, such as viruses and bacteria, mediated by cells and cytokines involved in the innate and adaptive immune responses (31). Human Leukocyte Antigens (HLA) are proteins encoded by the human MHC genes, which are the most highly polymorphic in the human genome. Individuals display between three and six different *HLA* alleles that present a variable distribution in the worldwide populations. The resulting HLA molecules’ variability affects the cellular immune response to peptides from human infecting-pathogens (32, 33). For instance, chronic viral infections can result if CD8+ or CD4+ T cells have difficulty identifying the HLA class I or II antigens on the cell surface or lower expression levels of the HLA molecules (34).

In patients with COVID-19, differences in the immune response of patients with mild and severe forms of the disease have been observed, including IgM and IgG levels (35). Also, a report considered the impact of the variation of the theoretical capacity for binding SARS-CoV-2 peptides to explain the HLA’s relation with the clinical heterogeneity of the disease (36). Therefore, this locus variability could explain differential risk susceptibility among populations considering the role of HLA molecules in the modulation of immune response to SARS-CoV-2 to identify risk subjects and the design of personalized therapy (37).

One study evaluated the *HLA* class I and II alleles in 82 Han individuals from Zhejiang with COVID-19. Authors reported that HLA-C*07:29 and -B*15:27 were found in a higher frequency among patients with COVID-19 than in previous analyzed controls, after correction with the Benjamini-Hochberg method. Other alleles also identified in different frequencies among compared groups, but with uncorrected tests, include HLA-C*07:29, -C*08:01G, -B*15:27, -B*40:06, -DRB1*04:06, and -DPB1*36:01 alleles, which were found more frequently among patients than in controls; and, -DRB1*12:02 and -DPB1*04:01 alleles, which were less common among individuals with COVID-19 than in the control group (38). In the Italian population, an investigation comprising 99 subjects found associated the HLA-DRB1*15:01, -DQB1*06:02, and -B*27:07 alleles with COVID-19 susceptibility (39); while an ecological study strongly suggests a permissive role of HLA-C*01 and B*44 towards SARS-CoV-2 infection across Italy (40). Meanwhile, the HLA-A*11:01, -B*51:01, and -C*14:02 alleles were related to the worst outcome among a Chinese population sample (41).

Regarding the severity of the disease, a study including 72 Spaniards with COVID-19 reported three *HLA* alleles associated with higher mortality (HLA-A*11, -C*01, and -DQB1*04) when the scores of Sequential Organ Failure Assessment (SOFA) and Acute Physiology And Chronic Health Evaluation II (APACHE II) were controlled (42). The HLA-DRB1*08 was correlated with mortality of COVID-19 in the Italian population, and the peptide binding prediction analyses showed that the allele was unable to bind any of the SARS-CoV-2 peptides with high affinity (43). The HLA-C*05 allele was also correlated with COVID-19 mortality in an ecological study (44).

Also, in a recent *in silico* analysis of the binding affinity between HLA class I molecules and all SARS-CoV-2 peptides, the HLA-B*46:01 allele was identified as a vulnerability biomarker due to low predicting binding sites. In contrast, the HLA-B*15:03 was considered a protector allele for showing the most significant capacity to present highly conserved SARS-CoV-2 peptides. The HLA-A*25:01 and -C*01:02 alleles were also related to a low predicted capacity for SARS-CoV-2 epitope presentations, whereas the highest predicted presentation capacity was observed for HLA-A*02:02 and -C*12:03 alleles (45). In agreement, another study using artificial neural networks identified the HLA-B*46:01 and HLA-A*25:01 as weakly binding alleles, while HLA-A*02:02 was one of the *HLA* class I alleles found to present a strong binding to virus selected peptides (46). Interestingly, HLA-A*02 alleles, among other class I and II alleles, were also identified as functional molecules for presenting SARS-CoV-2 peptides in a bioinformatic prediction study. In this same last report, an ecological study was also performed, and the HLA-DRB1*01 allele was found associated with COVID-19 fatality in a Mexican population; and, although the authors have addressed several limitations, the result must be taken with caution (47).

Nevertheless, other *in silico* analyses reported a possible association of HLA-A*02:01 with increased risk for COVID-19 and a lower capacity of this allele to present SARS-CoV-2 antigens in comparison to other *HLA* variants (48). These results seem to be contradictory compared to those previously mentioned, in which HLA-A*02 alleles were considered to have an adequate predicted capacity of antigens presentation. Therefore, the association should be taken with caution until the results of clinical studies were published.

Regarding *HLA* haplotypes, the study of regional frequencies for the most common Italian haplotypes reported that the HLA-A*01:01-B*08:01-C*07:01-DRB1*03:01 and HLA-A*02:01-B*18:01-C*07:01-DRB1*11:04 were correlated with COVID-19 incidence and mortality, suggesting risk and protection-related haplotypes, respectively (49). In an association study performed in a Sardinian population, the three-loci haplotype HLA-A*30:02-B*14:02-C*08:02 was more common among patients with COVID-19 (50).

**Table 1** shows examples of worldwide populations where the mentioned *HLA* alleles are frequently found. Nevertheless, it is crucial to consider the results of a recent publication in which the relevance of the HLA alleles’ homozygosity and heterozygosity was observed. The authors evaluated the synthesis of influenza virus proteins and RNA in lymphocytes from serologically HLA-homozygous or -heterozygous donors after the cells were exposed to the virus. They found that specific HLA-A and HLA-B-homozygous lymphocytes did not synthesize influenza virus RNA or protein after virus exposure, suggesting an intrinsic resistance to influenza virus infection in homozygous but not for HLA-heterozygous cells (52).

**Table 1:** *HLA* alleles associated with SARS-CoV-2 infection susceptibility.

*HLA* alleles	Populations in which the allele is commonly found ^a^
**High-risk**	
-A*25:01	Colombia Arhuaco.
-B*46:01	Chinese populations, Hong Kong Chinese, Malaysia Peninsular Chinese, Singapore Chinese, Taiwan Han Chinese, Thailand Northeast, USA Chinese, Vietnam Hanoi Kinh.
-C*01:02	American Samoa, Australian Kimberly Aborigine, Chinese populations, Colombian populations, Hong Kong Chinese, Japanese populations, Malaysia Peninsular Chinese, Mexico Chihuahua Tarahumara, Mexico Hidalgo, Mezquital Valley/Otomi, Mexico Zapotec, New Caledonia, New Zealand populations, Papua New Guinea populations, South Korea, Taiwanese populations, USA Asian, USA Hawaii Okinawa, Venezuela Perja Mountain Bari, Vietnam Hanoi Kinh, Bolivia/Peru Quechua, Costa Rica populations.
**Low-risk**	
-A*02:02	Cameroon Bamileke, Israel populations.
-B*15:03	Burkina Faso Rimaibe, Guinea Bissau.
-C*12:03	Azores Terceira Island, German populations, Greece, Italian populations, Lebanon Mixed, Pakistan Burusho, Papua New Guinea Wanigela Keapara, Poland, Portugal Azores Terceira Island, Spain populations, Sudan Mixed, China Jingpo Minority, Colombian populations.
**Mortality/severity**	
-A*11	Myanmar, China, Thailand, Taiwan, Japan, Spain, Mexico, South Korea, Mongolia, France, United Arab Emirates, Iran.
-B*51:01	Italy North, Japan, China, Oman, Armenia, Greece, China, Saudi Arabia, Switzerland Lugano, United Arab Emirates, Portugal, USA South Dakota Lakota Sioux and North American Native, Germany, Croatia, Serbia, Mexico Sonora Seri and Chihuahua, Romania, China Guizhou Province Miao.
-C*01	China Wuhan, Japan, India Kerala Hindu Pulaya, Brazil Parana Japanese, Scotland Orkney, Thailand Northeast, South Korea, Norway Sami, Peru Arequipa Mestizo, Vietnam Hanoi, Mongolia Oold, Myanmar Mon.
-C*05	United Kingdom, England, France, Spain, Wales, Venezuela.
-C*14:02	Japan Kyoto and Osaka
-DQB1*04	Mexico populations, Norway Sami, Venezuela Zulia Maracaibo Mixed, Brazil Guarani Nandeva, Papua New Guinea Highland, Ecuador Amazonia Mixed Ancestry, USA OPTN Hispanic, Russia Siberia Chukchi, Malaysia Perak Rawa.
-DRB1*08	Taiwan, Brazil, Mexico, Chile, Sudan, Peru, Burkina Faso, Argentina, India, Japan, Venezuela, Colombia.

a Representative populations with reported frequencies >0.10 are included. Data from Allele Frequency Net Database http://www.allelefrequencies.net/ (51).

#### Cytokine Genes

The cytokine storm is a complex process that has been difficult to define and delimit. However, it refers to an immune system gone awry and an inflammatory response flaring out of control, which is associated with infectious and noninfectious diseases with a wide variety of consequences in the organism (53). As has been mentioned before, the cytokine storm plays a crucial role in severe COVID-19 cases. SARS-CoV-2 produces the activation of various immune cells (e.g., macrophages, monocytes, dendritic cells), which leads to the secretion of several cytokines, including the pro-inflammatory cytokine IL-6 (54). This cytokine plays a central role in cytokine storm with anti-inflammatory and pro-inflammatory effects by promoting T-cell proliferation and B-cell differentiation, affecting vascular disease’s hormone-like properties, lipid metabolism, insulin resistance, mitochondrial activity, neuroendocrine system, and neuropsychological behavior (55).

High levels of IL-6 can activate the coagulation pathway and vascular endothelial cells but inhibit the myocardial function (56). In severe COVID-19 patients, an increase of IL-6 levels has been observed and related to the disease’s poor prognosis (57). Several gene variants in *IL6* (HGNC:6018) with differential cytokine expression and with different disorders have been reported. The rs1800795 (-174C) allele, as well as the promoter variant rs1800796 (-572C), have been associated with higher IL-6 plasma levels (58, 59) and with the risk of upper respiratory tract infections (60–62). Moreover, both *IL6* variants have been related to the prognosis of different disorders such as sepsis (63), coronary heart disease (64), and diabetes (65). A third variant (rs1800797) on the *IL6* promoter reported (66), and its role in studying the genetics of COVID-19 related-cytokine storm can be considered. In addition, seven variants in *IL6* (rs140764737, rs142164099, rs2069849, rs142759801, rs190436077, rs148171375, rs13306435) and five variants in *IL6R* (rs2228144, rs2229237, rs2228145, rs28730735, rs143810642) have been predicted to alter the expression and interaction of IL6 and IL6R which can be implicated in the pathogenesis of COVID-19 and its complications (67).

Genetic variants in the regulatory regions of other cytokines genes have also been reported (68). For instance, non-synonymous variants affecting the final proteins of TGF-β and IFN-α, as well as variants modifying the transcriptional activity of TNF-α, IL-10, and IL-2, have been described (68, 69). Several of these variants have been previously related to infectious disease susceptibility, cytokine storm, and venous thrombosis. Reported variants in cytokines genes associated with those events and their frequencies are shown in **Table 2**.

**Table 2:** Frequency of allelic variants in cytokine genes associated with infectious disease susceptibility and COVID-19 manifestations.

Cytokine gene	Variants studied	Allele frequency reference ^a^	Ref
**Infectious diseases susceptibility**			
*IL1B*	rs16944 g.4490T>C	European A= 0.3499 African A= 0.5726 East Asian A= 0.4692 South Asian A= 0.6000 American A= 0.5500	(70)
*IL17A*	rs2275913 g.4849G>C	European G= 0.6203 African G= 0.9508 East Asian G= 0.5069 South Asian G= 0.6200 American G= 0.7840	
*IL6*	rs1800795 g.4880C>G	European C= 0.4155 African C= 0.0182 East Asian C= 0.0010 South Asian C= 0.1390 American C= 0.1840	(60–62, 71)
*TNF*	rs1800629 g.4682G>A	European G= 0.8658 African G= 0.8805 East Asian G= 0.9415 South Asian G= 0.9470 American G= 0.9310	(61, 62)
**Venous thrombosis**			
*IL1B*	rs1143633 g.8890G>A	European C= 0.6660 African C= 0.8260 East Asian C= 0.4613 South Asian C= 0.7480 American C= 0.7090	(72)
*IL1R1*	rs3917332 g.102180064A>T	European A= 0.1938 African A= 0.0825 East Asian A= 0.0724 South Asian A= 0.1350 American A= 0.1540	
*IL1RN*	rs2232354 g.16866T>G	European T= 0.7962 African T= 0.9924 East Asian T= 0.9534 South Asian T= 0.7900 American T= 0.8310	
**Cytokine storm**			
*IL6*	rs1800796 g.4481G>C	European G= 0.9523 African G= 0.8971 East Asian G= 0.2093 South Asian G= 0.6050 American G= 0.7050	(58, 59)
	rs1800797 g.4456A>G	European A= 0.4076 African A= 0.0166 East Asian A= 0.0010 South Asian A= 0.1340 American A= 0.1840	(66)
*FCGR2A*	rs1801274 g.9541A>G	European A= 0.4891 African A= 0.4743 East Asian A= 0.7222 South Asian A= 0.5810 American A= 0.5490	(73)

a Data from 1000 genomes project (74).

In an Iranian population, genotypes of *IL1B* (HGNC:5992) rs16944 and *IL17A* (HGNC:5981) rs2275913 were associated with severe influenza A/H1N1 and B cases, while the frequencies of *IL10* (HGNC:5962) rs1800872 and *IFNL3* (HGNC:18365) rs8099917 variants were not found different among patients and controls (70). The *TNF* (HGNC:11892) rs1800629 variant has also been associated with variation in the corresponding cytokine and respiratory infections (61, 62).

Regarding the risk of venous thrombosis, 18 single-nucleotide variants in *IL1B* (HGNC:5992), *IL1RN* (HGNC:6000), *IL1R1* (HGNC:5993), and *IL1R2* (HGNC:5994), as well as 25 haplotypes, were evaluated in a case-control study including patients with deep vein thrombosis and controls. Authors found associated the *IL1B* rs1143633, *IL1R1* rs3917332, and *IL1RN* rs2232354 variants with different risks for venous thrombosis and an increased thrombotic risk for homozygous carriers of the *IL1RN* haplotype 5 GTGTA (rs3181052/rs419598/rs2232354/rs315952/rs315949) (72).

The Fc-gamma Receptors (FcγR) have been implicated in Fc-dependent cytokine release stimulation due to human leucocytes’ activation to secret various pro-inflammatory cytokines, as GM-CSF, IL-6, and IL-8 (75). The rs1801274 Fc fragment of IgG Receptor IIa (*FCGR2A*, HGNC:3616) gene was associated with severe pneumonia in patients with A/H1N1 infection. This variant produces a change of histidine to arginine at position 131 of the amino acid sequence. The frequency of homozygous individuals for p.His131 genotype was found to be increased in severe pneumonia patients (36.6%) in comparison to household contacts who did not develop respiratory illness (13.2%). Another gene reported in this study was the RPA Interacting Protein (*RPAIN*, HGNC:28641) and Complement C1q Binding Protein (*C1QBP*, HGNC:1243) (73).

Also, several *in vivo* and *in vitro* studies of influenza virus infection with lung damage due to cytokine storm have found a strong up-regulation on cytokine gene expressions, such as *IL6*, *IL8* (*CXCL8*, HGNC:6025), *CCL2* (HGNC:10618), *CCL5* (HGNC:10632), *CXCL9* (HGNC:7098), and *CXCL10* (HGNC:10637); as well as a differential expression of inflammasome genes *NLRP3* (HGNC:16400) and *IL1B* (HGNC:5992), cytokine genes *TNF* and *IFNB1* (HGNC:5434), and cytokine receptor genes *TNFRSF1B* (HGNC:11917) and *IL4R* (HGNC:6015) (53). An investigation found inborn errors of Toll-like receptor 3 (*TLR3*, HGNC:11849)– and interferon regulatory factor 7 (*IRF7*, HGNC:6122)–dependent type I IFN immunity related to life-threatening COVID-19 pneumonia. Although the genetic variants were only found in 3.5% of the studied patients, the results suggested that other IFN variants were probably implicated in the COVID-19 severity and the use of type I IFN as a potential therapeutic strategy in those patients (76). Likewise, a nested case-control study reported that *TLR7* (HGNC:15631) deleterious variants were found in 2.1% of severely affected males and none of the asymptomatic participants, and the corresponding functional gene expression analysis showed a reduction in the *TLR7* expression in patients compared with controls suggesting an impairment in type I and II IFN responses (77).

It is worth mentioning that wide inter-ethnic variability in cytokine gene variants’ frequencies (*IL2, IL6, IL10, TNF, TGFB1*, and *IFNG*) has been reported (68, 78). For instance, significant differences in *IL2* (HGNC:6001) alleles’ distribution among Africans, Caucasians, and Asians have been observed. Meanwhile, high expression alleles of *IL6* and *IL10* (HGNC:5962) have been more frequently found in Africans, Hispanics, and Asians, than Caucasians. Besides, low expression alleles of *IFNG* (HGNC:5438) have been more common among Asians than Caucasians (68).

### Variants in Coding Genes for Human Receptors of SARS-CoV-2

SARS-CoV-2 presents a high binding affinity to the ACE2 receptor allowing the virus’s entry to the host cell cytosol through acid-dependent proteolytic cleavage of the S protein, with a contribution of the TMPRSS2 and CTSL (21). Besides its role in SARS-CoV-2 infection, ACE2 acts as a negative regulator of the renin-angiotensin system and a facilitator of amino acid transport. The ACE2 system is a critical protective pathway against heart failure with reduced and preserved ejection fraction, including myocardial infarction and hypertension, lung disease, and diabetes mellitus. Unfortunately, the function of ACE2 is lost following the binding of SARS-CoV-2 (79).

Increased ACE2 receptor levels and the two proteases have been associated with identified risk conditions (e.g., increasing age, male gender, and smoking) of COVID-19 susceptibility and clinical outcome (21). Also, genetic variants of *ACE2* (HGNC:13557) that alter its transcriptional activity have been described (e.g., rs2285666, c.439+4G>A) (80, 81). An early study found higher allele frequencies of variants (e.g., rs143695310) associated with elevated expression of *ACE2* among East Asian populations, which may suggest a higher susceptibility to COVID-19 individuals from this region (82). A recent investigation has reported that genetic determinants of the highest expression of *ACE2* can be observed in South Asian and East Asian populations, while the lowest expression levels of *ACE2* were observed for Africans (83). Likewise, a genetic predisposition for the lowest *TMPRSS2* (HGNC:11876) expression levels was observed for Africans and the highest for East Asians. Moreover, significant differences in *TMPRSS2* expression levels among males and females were reported in the study (83).

Besides, variants with potential impact on the receptor stability have been reported. For instance, three common missense changes in *ACE2* (p.Asn720Asp, p.Lys26Arg, and p.Gly211Arg) were predicted to interfere with protein structure and stabilization, while other two variants (p.Leu351Val and p.Pro389His) has been predicted to interfere with SARS-CoV-2 spike protein binding (84). Likewise, a study using web-based tools reported several variants in genes that encode proteins related to the SARS-CoV-2 entry into the host cells: the already mentioned *ACE2* and *TMPRSS2*, as well as *TMPRSS11A* (HGNC:27954), *ELANE* (HGNC:3309), and *CTSL* (HGNC:2537). The authors found 48 variants in these genes with possible functional consequences, and some of them were reported to be shared among specific populations (85).

Nevertheless, the association results of the receptor variants with COVID-19 susceptibility remain controversial. For instance, Hou et al. found associated *ACE2* variants, such as p.Arg514Gly, in the African/African-American populations with cardiovascular and pulmonary conditions due to the alteration of the angiotensinogen-ACE2 interactions. Additionally, the authors identified variants in *TMPRSS2* (e.g., p.Val160Met, rs12329760) that could explain the COVID-19 susceptibility and some complication risk factors such as cancer and male gender (86). Meanwhile, a study with multi-scale modeling approaches in combination with sequence and phylogenetic analysis evaluated eight relevant variants located at the interaction surface of *ACE2* (i.e., rs961360700, rs143936283, rs146676783, rs759579097, rs370610075, rs766996587, rs73635825, and rs781255386). These SNPs are rare variants, except for European (non-Finnish) and African populations, and none of them would disrupt this receptor’s interaction with SARS-CoV-2 proteins (87).

Finally, the implication of an *ACE1* (HGNC:2707) deletion/insertion (D/I, intron 16) variant in the ACE2 expression and the COVID-19 clinical course was also proposed at the early stages of the pandemic (88). Nevertheless, later studies have reported that this variant could be related to the COVID-19 severity, but only if the patients’ hypertension status is considered (89, 90).

### Variants in Other Genes Related to COVID-19 Susceptibility and Severity

In addition to immune and SARS-CoV-2 receptors’ genes, variants in genes coding other proteins related to susceptibility and severity of COVID-19 have been identified. Recently, two independent genome-wide association studies (GWAS) had been performed among European populations (Italian and Spanish) (91) and individuals from the United States and the United Kingdom (92). In both cases, an association of *loci* 3p21.31 and 9q34.2 with COVID-19 severity were identified. The first study by Ellinghaus et al. reported the associations of *LZTFL1* (HGNC:6741) rs11385942, at locus 3p21.31, and *ABO* (HGNC:79) rs657152, at locus 9q34.2, with genetic susceptibility to COVID-19 (91). Meanwhile, Shelton et al. identified several non-genetic conditions as risk factors for hospitalization, and the genetic variants *LZTFL1* rs13078854 and *ABO* rs9411378 were associated with COVID-19 outcome severity and diagnostic, respectively (92). *LZTFL1* encodes the ubiquitously expressed protein leucine zipper transcription factor-like 1, and it is strongly expressed in human lung cells (91). Nevertheless, none of the publications can explain this gene’s role in the susceptibility or severity of COVID-19, but there are several genes nearby in the 3p21.31 locus that could plausibly be driving the association, including *SLC6A20* (HGNC:30927), *CCR9* (HGNC:1610), *FYCO1* (HGNC:14673), *CXCR6* (HGNC:16647), and *XCR1* (HGNC:1625) (92).

The role of *ABO* in COVID-19 susceptibility and clinical manifestations has been reported in genetic and non-genetic studies. Previous reports (93–95) and GWAS (91, 92) have observed a higher risk of COVID-19 infection among individuals with A group than other blood groups and a lower susceptibility for the O group. ABO blood group has been previously associated with infection susceptibility of other diseases such as influenza, malaria, schistosomiasis, and SARS-CoV. The hypotheses that blood groups can serve as receptors and/or co-receptors for bacteria, viruses, and parasites and that those blood antigens contribute to intracellular uptake, signal transduction, or adhesion have been stated (96). Besides, the idea that natural antibodies related to blood groups could contribute to the virus’s innate immune response has been proposed. Nevertheless, the ABO groups’ precise role in the SARS-CoV-2 infection mechanism still needs to be demonstrated (92).

Wang et al. also performed a GWAS among 332 Chinese patients and pedigree analysis. The authors reported the association with COVID-19 severity of the gene locus located in *TMEM189* (*PEDS1*, HGNC:16735)*–UBE2V1* (HGNC:12494), which is involved in the IL-1 signaling pathway. In the pedigree analysis, a potential monogenic effect of loss of function variants in *GOLGA3* (HGNC:4426) and *DPP7* (HGNC:14892) was suggested when authors looked for rare variants in families where a differential clinical outcome was observed among siblings (41). One more GWAS performed in 2,244 critically ill patients with COVID-19 from intensive care units in the United Kingdom found significant associations in several *loci*: in a gene cluster that encodes antiviral restriction enzyme activators *OAS1* (HGNC:8086), *OAS2* (HGNC:8087), and *OAS3* (HGNC:8088); near the gene that encodes tyrosine kinase 2 (*TYK2*, HGNC:12440); within the gene that encodes dipeptidyl peptidase 9 (*DPP9*, HGNC:18648); and in the interferon receptor gene *IFNAR2* (HGNC:5433) (97).

Patients with critical COVID-19 can present venous thromboembolism and/or systemic coagulopathies such as Disseminated Intravascular Coagulation (DIC) (13). This complication is characterized by the combined occurrence of activation of the extrinsic coagulation pathway and decreased activity of the protein C-protein S and Antithrombin (AT) inhibitory pathways, and it can be presented with excessive or inhibited fibrinolysis (98). DIC’s clinical and laboratory characteristics in COVID-19 are different from the typical presentation of these conditions, and a timely diagnosis is required to avoid the deterioration of pulmonary oxygen exchange (13). In this sense, a genetic marker that could predict coagulation complications could help to start appropriate treatment. For instance, the involvement of Mannose-Binding Lectin (MBL) and MBL-associated serine protease (MASP)-1/3 in coagulation has been reported, and its deficiency has been considered as a risk factor for DIC during sepsis complication; therefore, genetic variants producing a decrease of these proteins or their activity could be positively related with coagulopathies secondary to COVID-19 (99).

Besides, other genes with risk variants for DIC have been identified. In the anticoagulant pathways, variants in protein C gene (*PROC*, HGNC:9451), factor V Leiden (*F5*, HGNC:3542), and deficiencies of AT (*SERPINC1*, HGNC:775) have been related to an impaired function of the coagulation. While variants in the serpin plasminogen activator inhibitor 1 (*SERPINE1*, HGNC:8583) could impact the encoded protein levels, which is considered one of the main inhibitors of fibrinolysis, and it is related to DIC development. Additionally, variants in fibrinogen genes that promote the pro-coagulant pathways leading to microvascular thrombi formation in various organs have been described (98).

## Discussion

The present review provides an overview of different genes implicated or related to the susceptibility or severity of COVID-19. Nevertheless, with the information available to date, not everything has been resolved about the genetic involvement in COVID-19 susceptibility or severity, and new knowledge in the field has been continuously generated. Moreover, several consortia are dedicated to assessing the genetic determinants of COVID-19 in the worldwide population (30).

The COVID-19 presents a wide variability of clinical manifestations, from asymptomatic individuals to critical patients with fatal outcomes. Therefore the phenotype characterization probably represents the biggest challenge in COVID-19 genetic association studies of susceptibility and/or severity; mainly, to accomplish that selected subjects as controls had not presented the asymptomatic form of the disease. According to the reported studies, the comparison groups, co-variables adjustment, non-genetic factors, and asymptomatic individuals have great relevance in the association studies of genetic variants with COVID-19 susceptibility and clinical outcome.

Several *HLA* alleles have been associated with COVID-19 susceptibility and severity through various methodologies and in specific populations. In some cases, the *HLA* allele found related to the disease is shared between populations. For instance, the HLA-A*11 was reported in the investigations, including a sample of Chinese and Spanish populations, but it was not associated with Italian patients. Therefore, the interethnic variability in the *HLA* allele frequencies should be taken into account to identify the COVID-19 genetic marker. Moreover, the impact of SARS-CoV-2 genome variants in the host alleles associated must be assessed since the efficiency of antigens presentation by HLA molecules would be different according to the sites of the virus mutations (100, 101).

Current evidence highlights the relevance of cytokine storm in COVID-19 severity and several complications, including a fatal outcome. Genetic and non-genetic factors could explain the uncontrolled inflammatory response; therefore, the study of cytokine genes with adequate co-variables adjustment could lead to the identification of genetic markers related to COVID-19 outcome and the design and/or selection of personalized therapy. The cytokine storm’s early control is crucial to improving COVID-19 patients’ evolution (102).

According to *ACE2* and *TMPRSS2* variants, the African populations could have a lower susceptibility to COVID-19 than East and South Asians. Nevertheless, variants in both genes have not provided the genetic information regarding COVID-19 susceptibility as was expected. The 3p21.31 chromosome region and variants in the *ABO* gene recently identified in two different GWAS including diverse populations have been a relevant finding in the genetic study of COVID-19; however, further studies enlightening the role of the proteins encoded by the identified genes in the COVID-19 and their association in other populations is still warranted. Information about the penetrance of the risk alleles is required, and the inclusion of miRNAs in these studies could complement the genetic studies of COVID-19 susceptibility and its severity (103).

Herein we have presented several genetic variants reported to be associated with COVID-19 susceptibility and/or severity and others implicated in the biological pathway of the disease, considered relevant to include in subsequent clinical studies. Identifying genetic markers associated with the susceptibility or clinical outcome of COVID-19 could provide an essential contribution to the knowledge of this disease for the detection of susceptible individuals or populations and the design of therapeutic strategies (i.e., vaccine and pharmacologic treatment).

## Author Contributions

IF-G and RF-V contributed to the manuscript’s design, the figures production, and the writing of the manuscript. All authors contributed to the article and approved the submitted version.

## Funding

IF-G was supported by a Posdoctoral fellowship by the Consejo Nacional de Ciencia y Tecnología ESTANCIAS POSDOCTORALES POR MÉXICO EN ATENCIÓN A LA CONTINGENCIA DEL COVID-19. The allocated budget supports this work to research (RFV-HLA Laboratory, protocol number C53-20) from the Instituto Nacional de Enfermedades Respiratorias Ismael Cosio Villegas (INER).

## Conflict of Interest

The authors declare that the research was conducted in the absence of any commercial or financial relationships that could be construed as a potential conflict of interest.
